# Retention of work capacity after coronary artery bypass grafting. A 10-year follow-up study

**DOI:** 10.1186/1749-8090-4-6

**Published:** 2009-01-29

**Authors:** Ville Hällberg, Matti Kataja, Matti Tarkka, Ari Palomäki, the Working after CABG study group

**Affiliations:** 1 Department of Emergency Medicine, Kanta-Häme Central Hospital, Hämeenlinna, Finland; 2 Department of Internal Medicine, Hatanpää Hospital, Tampere, Finland; 3 National Public Health Institute, Helsinki, Finland; 4 Department of Cardio-Thoracic Surgery, Heart Center, Tampere University Hospital, Tampere, Finland

## Background

The major objectives in coronary artery bypass grafting (CABG) are restoration of normal functional capacity and quality of life as well as resumption of professional activity [1]. Factors predictive for return to work after CABG have been examined in various studies and are fairly well known. Return rates vary widely around the world, due to various factors such as different sick insurance systems, labour market conditions and sick listing traditions among physicians [2]. Only few studies have focused specifically on retention of work capacity after CABG.

In the earlier part of the Working after CABG (W-CABG) study we examined return to work and the length of continuation in a Finnish CABG patient population. According to the follow-up of patients remaining under 60 years, almost half returned to work successfully for a longer period. One fourth retired preoperatively and one fourth during the first postoperative year. After 5 years, pensioning of patients once successfully returned to work did not differ from that in the matched general population [3].

### Aims of the present study

In this paper we report postoperative continuation in work life among men and women aged less than 60. It was of special interest to evaluate the following aspects:

1) Which factors influence patients staying on?

Why do people who have resumed work after CABG retire prematurely?

2) What kind of model would help to estimate patients' possibilities of staying at work?

## Methods

### Material

The population of the W-CABG study has been described elsewhere [3]. Briefly, CABG was undertaken for 960 patients in Tampere University Hospital during a period of 18 months. The subjects of the present study comprised 141 consecutive patients aged less than 60 who had undergone CABG and were working one year postoperatively.

### Data collection

Study personnel checked hospital records after transfer to primary care. The follow-up checks were made with a detailed questionnaire on an average 21 months after the operation and 9.5 years after the operation. The reply percents were 89 and 83 of patients alive, respectively. Working status and other relevant information was obtained from all but two patients.

Employment status or date and reason for possible retirement were checked from the Finnish Centre for Pensions 10 years after the last operation [3]. Possible death, its date and diagnosis were checked from the Statistical Office of Finland. The study was approved by the Ethics Committee of Tampere University Hospital.

### Statistical methods

Categorical variables were tested using chi-square test and Wilcoxon rank test (Return to work vs. pre-, peri- and immediate postoperative medical data, education, type of work, preoperative pensioning, patients' social situation and lifestyle elements such as exercise habits and smoking).

#### Bayesian analysis

A stepwise Bayesian approach was used in multivariate analysis to determine posterior probabilities and likelihood ratios and to ascertain the sensitivity and specificity of the rule [4-8]. The model was calculated taking into account all patients under 60 and working one year after CABG.

#### Theory

The Bayesian approach states how posterior probability of diagnosis D given symptom × is calculated using the prior probability of diagnosis D and its absence. These show how common the diagnosis D and its absence are in the population. The probabilities of symptoms given the diagnosis and its absence are commonly estimated by their frequencies in learning material [6,7].

In the case of a two-valued outcome, the Bayesian approach is commonly developed to give a **likelihood ratio **(probability of × given diagnosis D divided by probability of × given absence of diagnosis D). The likelihood ratio (L) of a set of observed symptoms or properties xi is written as the product of the probability ratios of each xi, also known in epidemiology as risk ratios.

The posterior probability of a given set of observations is calculated by dividing the likelihood ratio by "one plus likelihood ratio". Percentage is obtained by multiplying by 100.

#### Derivation of indices

One of the authors (MK) has developed an optimization procedure to facilitate the Bayesian analysis. The program goes through all variables given by the parameters and selects first only one variable which will best predict the selected outcome, staying at work over the median in our study. The program then goes through all the remaining variables and selects as second variable that which will best predict the outcome together with the first variable.

Adding one variable after another to the model, the program goes through all the variables. If there are variables which correlate with each other the program selects the best predictor and ignores others. The optimum is the number of variables which together give the smallest failure rate (maximum sum of sensitivity and specificity) in the study group. Usually the optimum does not include all given variables. Ultimately the program provides the best combination of variables which will most accurately predict the outcome.

The program gives the critical area for the total risk index, where the numbers of false-negative and false-positive results are minimal. It also calculates a Receiver Operating Characteristics (ROC) curve. It gives a graphic representation of the relationship between true-positive and false-positive rates and can be used to study the effect of changing the decision rule. The area under the ROC curve is commonly used to measure the predictive power of a statistical model [8].

## Results

### Study population

The study population comprised 141 CABG patients, 12 women and 129 men who were working one year postoperatively. Their demographic data and work classification are shown in Table 1. Clinical characteristics and severity of coronary heart disease are compiled in Table 2. Besides CABG an aortic valve replacement was carried out for one patient (male, age 52 years). Four patients had been pensioned before the operation and returned to work after CABG, 137 had been preoperatively at most on short-term sick pay.

**Table 1:** Demographic data and job classification at the time of CABG

	**Female (n = 12)** **%**	**Male (n = 129)** **%**	**All (n = 141)** **%**
**Age**			
< 50	33	41	40
50 – 54	33	35	35
55 – 59	33	24	25
			
**Education**			
Less than high school	80	68	69
High school graduate	0	22	20
College graduate	20	10	11
			
**Job classification**			
Manual worker	25	46	44
Employee lower level	12	17	17
Employee higher level	25	16	17
Farmer	0	5	4
Employer or entrepreneur	38	16	18

**Table 2:** Clinical characteristics and severity of coronary heart disease before CABG

	**Female (n = 12)** **%**	**Male (n = 129)** **%**	**All (n = 141)** **%**
**Concomitant diseases**			
Hypertension	83	33	38
Hypercholesterolemia	90	91	91
Diabetes mellitus	25	4	6
Previous MI	70	61	62
Previous TIA or stroke	0	3	3
Intermittent claudication	0	4	3
			
**Smoking**			
Previous	30	60	58
Current	20	15	16
			
**Cardiac function**			
EF 35 to < 50%	9	7	7
EF < 35%	0	3	3
NYHA II	17	20	20
NYHA III	58	64	63
NYHA IV	25	16	17
			
**Severity of CHD**			
Three-vessel disease	33	48	47
Left main disease	8	10	10
Elective operation	91	86	87
Urgent operation	9	12	11
Emergency operation	0	2	2

(MI = myocardial infarct, TIA = transient ischemic attack, EF = ejection fraction, NYHA = angina pectoris symptoms according to New York Heart Association, CHD = coronary heart disease)

### Continuation at work after CABG

**Figure 1:**
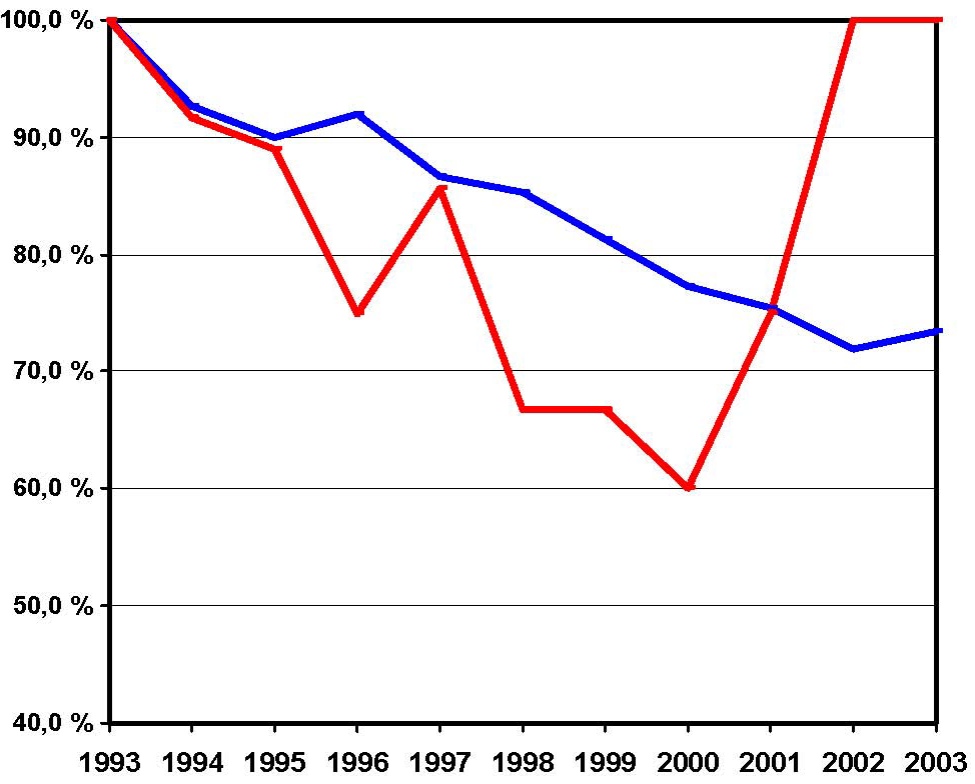
**Postoperative employment status at the end of each calendar year**. A 10-year follow-up of originally 141 patients, who were working one year postoperatively and remained under 60 during the follow-up. Deceased persons, altogether eight, are excluded. X-axis: Calendar years. Y-axis: Percentages of patients working at the end of each calendar year. Blue line: men, red line: women

The yearly employment status (full-time or part-time work) gradually decreased in men from 100% to 85% (n = 95) in five years. Correspondingly, it was 73% (n = 49) ten years postoperatively. The results for women were affected by their small number (n = 7 five years and n = 3 ten years postoperatively) (Figure 1). Altogether 76% of patients aged < 60 years were still working 10 years after CABG.

All 141 patients were younger than 60 one year after CABG. Twenty-six of them (17%) retired on disability pension before the age of 60, the main reason being cardiac condition in 16 cases. Ten patients retired for other reasons (stroke, pulmonary embolism, psychiatric and musculoskeletal disorders). During the follow-up, a total of 15 CABG patients retired to adjusted old-age pension at the age of 57 – 60 mainly for non-medical reasons. Half of them retired 1 to 2 years after CABG.

### Model of continuation at work

#### Results of univariate analysis

In univariate analysis, the three most significant factors (p < 0.001) affecting staying on were patients' age at the time of the operation, preoperative working status and patients' height.

Other factors describing different aspects of patients' medical condition and social situation evinced some lesser correlation with continuing to work. Some were related to preoperative conditions such as kidney function, some others to the extent of cardiovascular disease or postoperative well-being (NYHA class, nitrogen use and subjective well-being when compared to preoperative condition). Patients' size was shown not only by patients' height but also by sex and weight. Other factors described diabetic burden (body mass index or diagnosed type 2 diabetes mellitus), overall physical activity (daily walking distance, walking restrictions and frequency of participation in household work) and social situation, e.g. patients' marital status.

#### Results of multivariate analysis

When forming the model for remaining at work by multivariate Bayesian analysis, we compared several patterns of the factors presented above. Finally, patient's younger age at the time of CABG was selected as the best predictor for continuation at work. The best balanced and most useful model also included the following factors: postoperative cardiac symptoms, patients height, marital status, diabetes mellitus and participation in household work (Table 3).

**Table 3:** The probability of remaining at work for at least three, six and nine years

**Variable**	**Over 3 years**	**Over 6 years**	**Over 9 years**
	**(n = 119)**	**(n = 92)**	**(n = 59)**
**1. Age at the time of CABG**			
≤ 50	**LR = 6.54**	**LR = 3.25**	**LR = 2.42**
50 – 54	**LR = 3.80**	**LR = 1.24**	**(LR = 0.20)**
≥ 55	**LR = 0.68**	**LR = 0.37**	**(LR = 0.01)**
			
**2. Postoperative cardiac symptoms**			
No	**LR = 1.11**		**LR = 1.08**
Yes	**LR = 0.51**		**LR = 0.51**
			
**3. Height**			
< 175	**LR = 0.59**	**LR = 0.58**	
175 – 179	**LR = 1.43**	**LR = 1.80**	
180-	**LR = 6.29**	**LR = 2.13**	
			
**4. Being married**			
No	**LR = 3.51**	**LR = 1.60**	**LR = 1.14**
Yes	**LR = 0.88**	**LR = 0.93**	**LR = 0.98**
			
**5. Diagnosed DM**			
No	**LR = 0.92**	**LR = 1.03**	**LR = 1.04**
Yes	**LR = 2.59**	**LR = 0.80**	**LR = 0.70**
			
**6. Participation in household work**			
Daily		**LR = 1.25**	**LR = 1.26**
Less often		**LR = 0.58**	**LR = 0.51**
			
**Precision of the model**			
**False-Positive**	**10**	**18**	**15**
**False-Negative**	**9**	**17**	**13**
**Sensitivity**	**55%**	**63%**	**82%**
**Specificity**	**92%**	**82%**	**78%**
**Correct predictions**	**86%**	**75%**	**80%**

In this model, the individual probability of remaining at work (calculated one year postoperatively) is as follows:

The patient's likelihood ratio (**LR**) of each of six characteristics is multiplied. The greater LR, the better is the possibility of remaining at work. If the total LR is greater than 1.00, the probability of remaining at work is more than 50% for the given time (3 y, 6 y, or 9 y). Patients in age groups marked in parenthesis may remain at work for over the age of 60.

LR may be expressed as probability as follows: p = 100 * LR/(1+LR).

As an example, we have a 52 year old married patient, who does not participate in household work. He/she is 176 cm of height and is not suffering from diabetes or postoperative cardiac symptoms. Hence, the total likelihood ratio of remaining at work is 1.24 × 1.80 × 0.93 × 1.03 × 0.58 = 1.24. According to the model [100 * LR/(1+LR)], we get 55% probability of remaining at work for at least six years. If our patient would be an active person for example participating into the household work, his/hers total likelihood ratio of remaining at work for at least six years would increase to 1.24 × 1.80 × 0.93 × 1.03 × 1.25 = 2.67 and thus his/hers probability were 73%.

**Figure 2:**
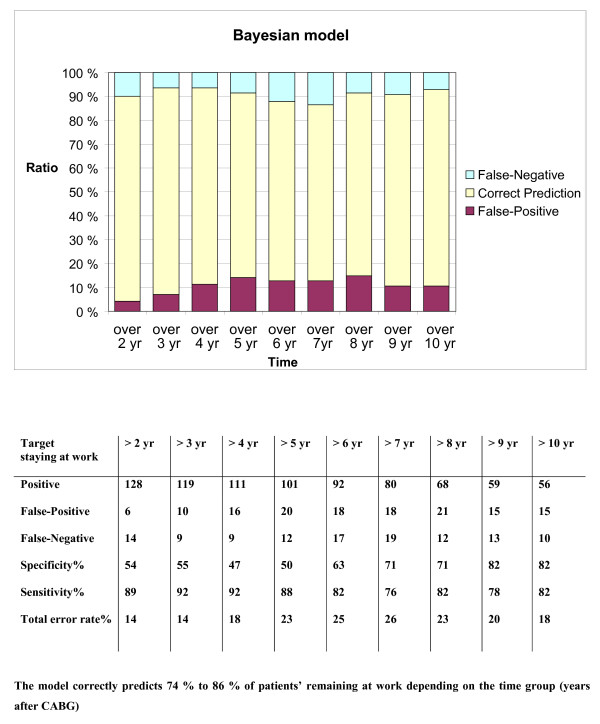
**The ability of the model to predict remaining at work**.

**Figure 3:**
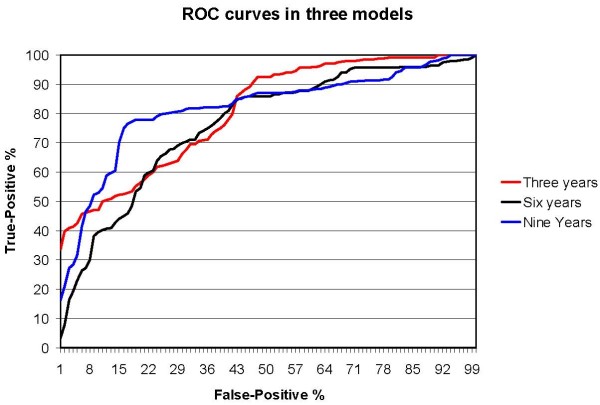
**ROC curves of the ability of the model to predict remaining at work**.

The ability of the model to predict continuation was analyzed in annual time intervals throughout the 10-year postoperative follow-up. In this population the total error rate was 14% to 26% (Fig 2). The corresponding ROC curves are shown in Fig. 3.

## Discussion

### Principal findings

In this paper we present a model for estimating patients' possibilities to remain in work after CABG. We also report that 73% of men who returned to work postoperatively and were aged less than 60 years were still working 10 years later. The number of women was not large enough for scrutinized statistical comparison, but their continuation at work resembled that of men.

### Strengths and weaknesses of the study

To the best of our knowledge this study represents the longest follow-up of CABG patients' retention of work capacity. We analyzed data on all consecutive patients operated over a period of 18 months. Their urban and rural domiciles comprise more than 20% of the inhabitants of the country, thus well representing the Finnish population.

The study was commenced with a retrospective analysis of patient records. In addition, written patient questionnaires and telephone contacts were used. Follow-up data on pensioning and mortality were verified from national archives, which in Finland are particularly precise. Even after these measures some postoperative data, for instance in patient questionnaires, were missing.

Our study population was only 141 patients. In general, relevant long-term follow-up data on CABG patients' working status are somewhat difficult to obtain by reason of the relatively high age of the patients operated. Especially the female population was very small, and the interpretation of its postoperative work activity must be made very carefully. We therefore treated men and women as one group when forming the model. On the other hand, patients' uniformity in respect of age and burden of diseases made it easier to make significant calculations also from a fairly small material.

When building the model, possibly relevant parameters had first to be selected by univariate analysis. At this stage some, again, had to be left out owing to insufficiency of material. Some factors were not selected due to their low prevalence in the population studied. Of variables describing different aspects of the same feature, only the best was selected for the model.

The Bayesian approach in multivariate analysis was used in view of its ability to analyze partly incomplete material from questionnaires. In this study it also seemed to be more illustrative than logistic calculations based on an iterative process.

### Retirement before the age of sixty

Three quarters of the patients less than 60 were in working life ten years after CABG (Fig. 1). The difference seen between genders was evidently induced by the small number of women returning to work postoperatively and working until the age of 60.

Some of the retired patients had evident worsening of cardiac disease after the initial CABG. However, the majority of patients in this group were pensioned off because of a wide range of postoperative problems not necessarily related to coronary insufficiency. Of retirees aged less than 60 who resumed work 27% were pensioned during the first follow-up year. This may reflect the early retirement and disability pensioning system in Finland, which favours working attempts after CABG before obtaining a pension.

### Results in relation to other studies

Only a few published studies have evaluated continuation at work over a longer follow-up. None has introduced a model for remaining in work. However, it is possible to make some comparisons with shorter follow-up studies of two to five years which describe retention of working capacity after CABG. Skinner et al. have introduced a British material of originally 343 CABG patients [9]. At five-year follow-up the most important factors for continuation were younger age at the time of operation, male sex, preoperative working and freedom from postoperative angina symptoms. Hlatky et al. have presented an American material of 409 patients with either CABG or PCI [10]. Long-term employment did not differ significantly between these groups. The authors also found that predictors of longer working postoperatively were younger age at the time of the operation and preoperative full-time work, but also a single private source of medical insurance, which would indicate the importance of social factors. Left ventricular function appeared as a single medical factor. In the RITA trial [11], over 1000 patients were randomized either to CABG or PCI. Factors predicting working two years postoperatively were positive preoperative working status, younger age and male gender.

In the present study the model of continuation in work included patients' age and height, postoperative cardiac symptoms, diabetes, patients' matrimonial status and participation in household work.

In previous CABG studies age has been the most important predictor of continuing at work. As in the present study absence of postoperative cardiac symptoms was important in remaining at work also in a study by Skinner et al. [9]. Cardiac condition was also found in an earlier Finnish material to be the most important reason for not working five years postoperatively [12]. Patient's height is closely related to sex, which has been found to be a predictor of return [13]. Also diabetes mellitus has emerged as a negative predictor for return but it is also associated with a poorer long-term outcome after CABG [14,15]. In this context it is quite logical that these were selected for the present model of resumption.

In our model it would appear that diabetes favours working for three to five years postoperatively. This is coincidentally caused by the small number of diabetics in this group (one patient).

Finland, like many other European countries, has a national medical insurance system and thus insurance issues did not affect remaining at work. Probably for the same reason type of work likewise did not influence remaining at work.

Being married often constitutes a form of social and financial security. Vice versa, being unmarried might accentuate the importance of daily work as both social and financial protection. Participation in household work was in our material associated with other physical activities such as daily walking distance and exercise habits (data not shown). This may thus be considered as one dimension of physically active life. Preoperative working status was not selected into our model, since almost all patients were working or only on short-term sick leave preoperatively.

## Conclusion

In this part of the W-CABG study, we have analyzed patients' postoperative continuing to work. Age and height, postoperative cardiac symptoms, matrimonial status, diabetes mellitus, and participation in household work were selected into the final model. In the ten year follow-up, this model predicted 80% of continuation at work after coronary artery bypass grafting. Correspondingly, the most important single reason for premature retirement was found to be cardiovascular disease.

Our results encourage medical professionals to effectively treat the postoperative cardiac problems and diabetic burden of CABG patients as well as to activate them not only to undertake household work but also all kinds of social and physical activity.

## Competing interests

The authors declare that they have no competing interests.

## Authors' contributions

VH conceived of the study, and participated in its design and coordination and drafted the manuscript. MK performed the statistical analysis and helped to draft the manuscript. MT participated in the design and coordination of the study and revised it critically. AP participated in the design and coordination of the study and helped to draft the manuscript. VH and AP wrote the final manuscript, which was read and approved by all authors.
